# A Meta-Analysis of Folic Acid in Combination with Anti-Hypertension Drugs in Patients with Hypertension and Hyperhomocysteinemia

**DOI:** 10.3389/fphar.2017.00585

**Published:** 2017-08-31

**Authors:** Wen-Wen Wang, Xin-Shi Wang, Zeng-Rui Zhang, Jin-Cai He, Cheng-Long Xie

**Affiliations:** ^1^The Center of Traditional Chinese Medicine, The Second Affiliated Hospital and Yuying Children's Hospital of Wenzhou Medical University Wenzhou, China; ^2^Department of Neurology, The First Affiliated Hospital of Wenzhou Medical University Wenzhou, China

**Keywords:** folic acid, enalapril, hypertension, antihypertensive, hyperhomocysteinemia, meta-analysis

## Abstract

Folic acid is generally used to lower homocysteine concentrations and prevent stroke and cardiovascular disease (CVD) at present. However, the efficacy of therapies that lower homocysteine concentrations in reducing the risk of CVD and stroke remains controversial. Our objective was to do a meta-analysis of relevant randomized controlled trials (RCTs) to evaluate the efficacy of folic acid supplementation among patients with hypertension and Hyperhomocysteinemia (HT/HHcy). We included RCTs examining the effects of folic acid plus antihypertensive therapy compared to antihypertensive alone. Weighted Mean Difference (WMD) and Relative risk (RR) were used as a measure of the effect of folic acid on the outcome measures with a random effect model. Sixty-five studies including 7887 patients met all inclusion criteria. Among them, 49 trials reported significant effect of combination therapy for reducing SBP (systolic Blood Pressure) and DBP (Diastolic Blood Pressure) levels compared with antihypertensive alone (WMD = −7.85, WMD = −6.77, respectively). Meanwhile, folic acid supplementation apparently reduced the level of total homocysteine (WMD = 5.5). In addition, folic acid supplementation obviously reduced the risk of cardiovascular and cerebrovascular events (CVCE) by 12.9% compared with control groups. In terms of the stratified analyses, a bigger beneficial effect was seen in those RCTs with treatment duration of more than 12 weeks, a decrease in the concentration of total homocysteine of more than 25%, with folic acid fortification. Our findings indicated that folic acid supplementation was effective in the primary prevention of CVCE among HT/HHcy patients, as well as reducing the blood pressure and total homocysteine levels.

## Introduction

Prospective previous researches had confirmed a solid, graded, and independent positive association between blood pressure and risk of stroke and cardiovascular disease (CVD) (Chobanian et al., 2003). The latest epidemiological data showed that hypertension is the biggest single contributor to the global burden of disease, leading to approximately nine million deaths each year (Lim et al., 2012), which was largely mediated through CVD and stroke (Poulter et al., 2015). Furthermore, the numbers of subjects suffered from hypertension and the morbidity of high blood pressure worldwide were predicted to increase over the next decade (Kearney et al., 2005). What is more, hyperhomocysteinemia has also emerged as an independent risk factor for CVD and stroke. A recent meta-analysis showed that folic acid supplementation to lower total homocysteine (tHcy) concentrations could effectively reduce the risk of stroke in primary prevention (Wang et al., 2007). Meanwhile, it was interesting to note that several carefully conducted studies to date have showed hyperhomocysteinemia often accompany with hypertension in many experimental models, as well as in patients with primary hypertension, especially systolic blood pressure (SBP) (Lim and Cassano, 2002; Huo et al., 2015). In addition, some randomized, placebo-controlled trials (RCTs) of tHcy-lowering therapy had shown that such intervention was associated with a decrease in blood pressure (Mangoni et al., 2002). However, although Hyperhomocysteinemia associated hypertension patients (HT/HHcy) are common in clinic, the precise mechanism by which tHcy results in vascular dysfunction and contributes to hypertension are largely unknown (Sen et al., 2010). To date, several mechanisms have been proposed. Hyperhomocysteinemia related changes in vascular biology such as increased arterial stiffness, vascular smooth muscle cell proliferation and endothelial dysfunction could easily lead to an increase in blood pressure (Stehouwer and van Guldener, 2003).

The evidence from observational studies suggested that HT/HHcy levels were associated with increased danger of CVD and stroke compared with hypertension alone (Wang et al., 2007). Stanger and colleagues displayed that tHcy lowering may have intrinsic vasoprotective effects principally independent of folic acid supplementation (Stanger et al., 2002). Two meta-analyses reported that folic acid administration may be good for CVD prevention in patients with kidney disease, especially in patients without a history of grain fortification with folic acid, with lower percent baseline diabetes (Jardine et al., 2012; Qin et al., 2013). In addition, folic acid supplementation was effective in stroke prevention in populations with no or partial folic acid fortification (Huo et al., 2012). On the contrary, Zhou et al reported that folic acid did not impact on the incidence of CVD, stroke or all-cause mortality based on the 16 RCTs (Zhou et al., 2011). Taken together, multiple clinical trials had now been performed investigating folic acid administration for the prevention of CVD and stroke outcomes in HT/HHcy patients. However, there were still no meta-analysis concerns on tHcy-lower therapy in HT/HHcy. Our purpose was thus to do a meta-analysis focusing on efficacy of folic acid plus antihypertensive combination therapy among patients with HT/HHcy.

## Materials and methods

### Search strategy

We used a familiar search strategy as previously published study (Xie et al., 2014). We carried out a comprehensive and independent literature search of six databases of PubMed; Cochrane Central Register of Controlled Trials (CENTRAL); Google scholar; Wanfang database and Chinese National Knowledge Infrastructure (CNKI) and VIP information database. The publication time is from January 1990 of each database up to December 2016 for all English and Chinese language publications. To identify any other relevant studies, we manually scanned the reference lists of identified trials and review articles. The search terms were “hypertension,” “blood pressure,” “hyperhomocysteine,” and “homocysteine.”

### Inclusion and exclusion criteria

Clinical trials were eligible for inclusion if satisfy the following criteria:

1. Study design: RCTs.
2. Subjects were demanded to have a clinical diagnosis of idiopathic hypertension and also a laboratory diagnosis of hyperhomocysteinemia without a history of stroke or myocardial infarction. A priori definition of hyperhomocysteinemia and hypertension should have been identified. In this study, most trials based on the Chinese guidelines for management of hypertension (GPMH) (Liu and Writing Group of Chinese Guidelines for the Management of Hypertension, 2011), using SBP ≥ 140/DBP ≥ 90 mmhg and tHcy >10 umol/L.
3. Conducted to evaluate the combination of folic acid and antihypertensive compared with antihypertensive alone.
4. Have adequate data such as blood pressure, tHcy levels or cardiovascular and cerebrovascular events (CVCE) as the outcome measures. Among them, definition of CVCE is including angina, carotid artery stenoses, myocardial infarction, stroke, transient ischemic attack.

### Data extraction and quality assessment

The following data in the primary trials were extracted: first author's name, year of publication, diagnostic criteria of hypertension and hyperhomocysteinemia, basic data (age and sex), intervention, dosage of folic acid, treatment duration and outcome measures. In the event of outcomes were presented from the studies at different time points, we extracted data from the last time point. The validity of RCT was evaluated for risk of bias using the Cochrane Collaboration's tool (Higgins et al., 2011). The tool distinguished trials according to six domains; sequence generation, allocation concealment, blinding, missing data, selective reporting and other biases.

### Statistical analysis

We utilized a random effects rather than a fixed effects model due to this takes into account heterogeneity between multi-trials, as well as many experts consider the random effects model to be a more natural choice than fixed effects in medical decision making contexts (Ades et al., 2005). For the assessment of heterogeneity, the I^2^ statistic was used. For studies in which more than one folic acid supplementation regimen existed, we reported the high dosage folic acid both before and after the intervention. Publication bias was assessed by Begg's test. All the analyses were done with Revman software, version 5.0.

## Results

### Results of the search and study characteristics

**Figure 1:**
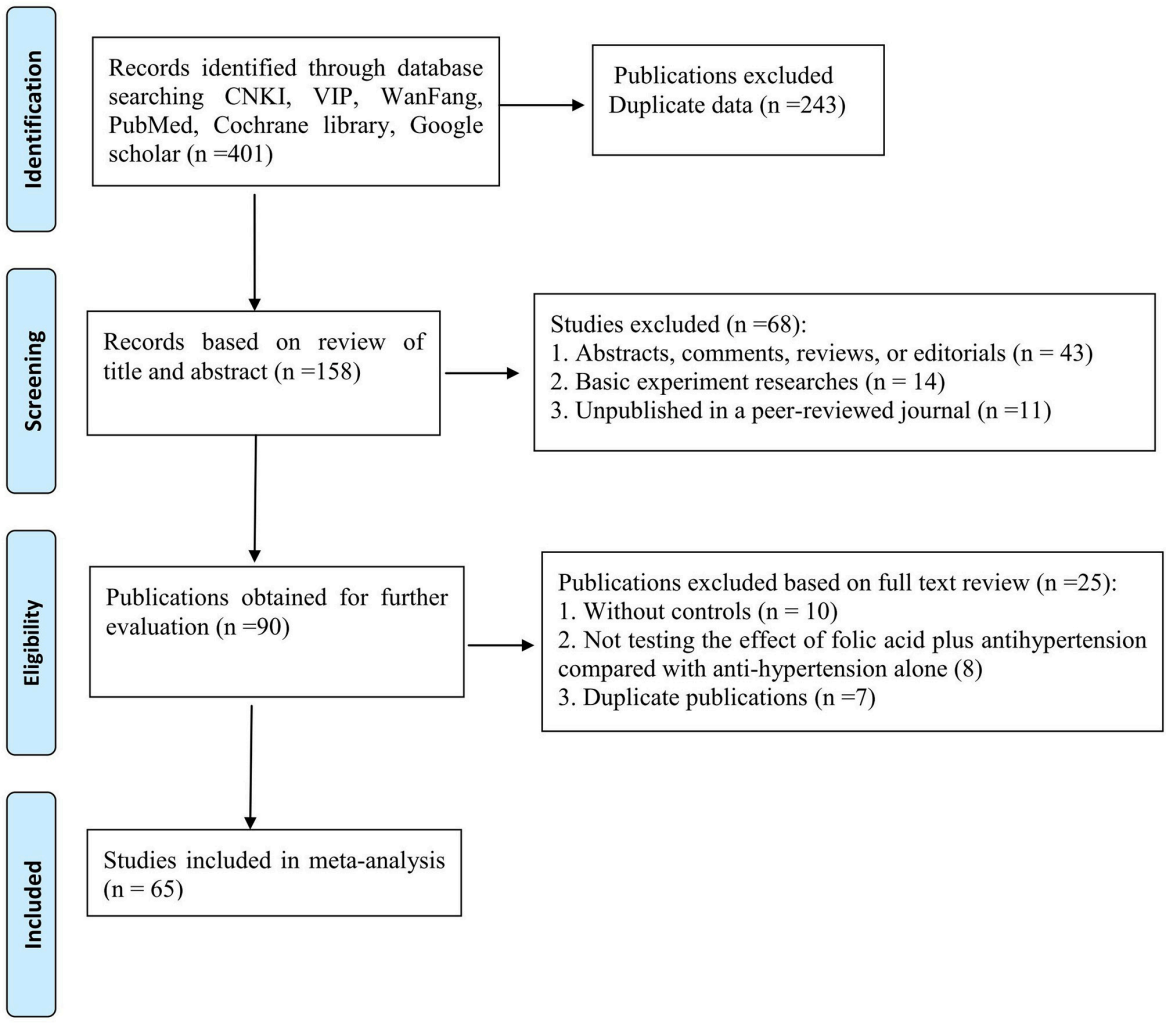
PRISMA 2009 Flow Diagram.

Our final analysis included 65 RCTs (Figure 1) and all trials were reported between 2012 and 2016 (Full citation details of included studies are provided as Supplemental Material I). The basic characteristics of the included trial participants and risk bias of these RCTs were presented in Table 1. Sixty-five RCTs, with a total of 7,887 subjects, met the inclusion criteria and were included in the meta-analysis. Of whom, 3,972 were randomized to folic acid plus antihypertensive groups, and 3,915 were randomized to antihypertensive alone groups. The number of subjects included in this review ranged from 50 to 320 subjects. Moreover, the time of follow up was ranged from 8 weeks to 18 months. The interventions were primarily based on daily oral folic acid supplementation in doses of between 0.4 mg and 10 mg plus antihypertensive therapies. Among them, 40 studies used 0.8 mg of folic acid; 13 studies utilized a daily dose of 0.4 mg of folic acid; 5 studies adopted 5 mg of folic acid in the treatment groups; a daily oral dose of 10 mg of folic acid only in the 1 study; remaining 6 studies did not show the dose of folic acid, respectively. Co-interventions included Enalapril in 54 trials, amlodipine + benazepril in two studies, remaining 9 studies did not show the drug. Treatments duration of all studies is including 2 weeks (2 studies), 4 weeks (1 study), 5 weeks (1 study), 6 weeks (2 studies), 8 weeks (22 studies), 3 months (19 studies), 4 months (1 study), 6 months (8 studies), 10 months (1 study), 12 months (4 studies), and 18 months (1 study), respectively, and remaining 3 studies did not report the treatments duration. Therefore, a median treatment duration is 3.8 months. In terms of outcome measure, SBP and DBP as the outcome measure were observed in 49 studies; The Hcy levels were observed in 60 studies; CVCE was reported in 22 studies. The risk of bias of these trials ranged from 1 to 4. Of whom, 15 studies got 1 point; 40 studies got 2; 9 studies got 3; 1 study got 4, an average risk of bias score is 1.9. Fourteen studies described the method of randomization, only five studies showed the blinding of participants. Therefore, all of these included trials were supposed to have a high risk of bias.

**Table 1:** Basic characteristic of included studies.

**Study**	Criteria (blood pressure/Hcy)^*^	**Basic data: M/F(n); age**		**Pre-treatment: Hcy (umol/L); BP (SBP/DBPmmHg); Intervention**		**Outcome measures**	**Risk bias**
1. Liu, 2012	180 > SBP ≥ 140;110 > DBP ≥ 90 mmhg Hcy ≥ 15 μmmol/L	162/158 (320), 52.33 ± 5.94		12.30 ± 4.32, NR Folate 0.4 mg + Enalapril 10 mg/d (*N* = 160)	15.22 ± 4.55, NR Enalapril 10 mg/d for 6 mo (*N* = 160)	1.CVCE 2.Hcy	1
2. Liu, X. 2012	SBP ≥ 140/DBP ≥ 90 mmhg Hcy ≥ 10 μmmol/L	26/20 (46) 67.6 ± 11.8	23/17 (40) 66.2 ± 9.9	15.2 ± 5.1, 138.3 ± 10.7/77.4 ± 7.8 Folate 0.8 mg + Enalapril 10 mg/d (*N* = 46)	17.2 ± 5.8, 135.6 ± 12.1/86.5 ± 11.4 Enalapril 10 mg/d for 12 w (*N* = 40)	1.SBP, DBP 2.Hcy	2
3. Lu, 2012	SBP ≥ 140/DBP ≥ 90 mmhg Hcy ≥ 12 μmmol/L	56/51 (107) 76.2 ± 5.3	50/47 (97) 75.7 ± 5.1	13 ± 0.6,151.6 ± 5.5/93.4 ± 4.2 Folate 10 mg + anti-hypertension therapy (*N* = 107)	13.1 ± 0.7, 150.5 ± 5.2/92.6 ± 4.5 Anti-hypertension therapy for 72 w (*N* = 97)	1.Hcy, CVCE 2.SBP, DBP	2
4. Meng, 2012	180 > SBP ≥ 140;110 > DBP ≥ 90 mmhg Hcy ≥ 15 μmmol/L	56/33 (89), 52.6 ± 7.2		16.3 ± 2.1, NR Folate 0.8 mg + Enalapril 10 mg/d (*N* = 45)	19.4 ± 3.2, NR Enalapril 10 mg/d for 8 w (*N* = 44)	1.Hcy 2.SBP, DBP	3
5. Shen, 2012	SBP ≥ 140/DBP ≥ 90 mmhg Hcy ≥ 10 μmmol/L	31/19 (50), 62.4 ± 5.2		20.32 ± 4.71, 151.58 ± 10.46/93.71 ± 8.53 Folate 0.8 mg + Enalapril 10 mg/d (*N* = 25)	21.11 ± 5.02,152.22 ± 9.85/92.85 ± 8.95 Enalapril 10 mg/d for 6 mo (*N* = 25)	1.Hcy 2.SBP, DBP, CVCE	2
6. Hu, 2013	SBP ≥ 140/DBP ≥ 90 mmhg Hcy ≥ 10 μmmol/L	77/63 (140), 58.2 ± 8.3		25.26 ± 5.62,157.25 ± 10.63/97.33 ± 7.82 Folate + Enalapril 10–20 mg/d (*N* = 70)	24.62 ± 5.76,154.83 ± 10.56/95.27 ± 7.82 Enalapril 10–20 mg/d for 2 mo (*N* = 70)	1.Hcy 2.SBP, DBP	3
7. Li, 2013	NR, Hcy ≥ 12 μmmol/L	61/39 (100), 59.0 ± 9.4		21.48 ± 3.46, NR Folate 0.8 mg + Enalapril 10 mg/d (*N* = 50)	21.54 ± 3.45, NR Enalapril 10 mg/d for 6 mo (*N* = 50)	1.Hcy, IMT 2. CVCE	2
8. Mao, 2013	SBP ≥ 140/DBP ≥ 90 mmhg Hcy ≥ 10 μmmol/L	48/52 (100), 62.4 ± 5.3		13.8 ± 12.3, 154.2 ± 10.3/93.4 ± 8.2 Folate 0.8 mg + Enalapril 10 mg/d (*N* = 50)	13.8 ± 12.1,152.4 ± 10.8/94.2 ± 8.6 Enalapril 10 mg/d For 8 w (*N* = 50)	1.Hcy, AE 2.SBP, DBP	2
9. Qin, 2013	SBP ≥ 140/DBP ≥ 90 mmhg Hcy ≥ 10 μmmol/L	46/22 (68), 52.0 ± 17.0		19.48 ± 3.42,152.28 ± 14.25/111.43 ± 5.48 Folate 0.8 mg + Enalapril 10 mg/d (*N* = 34)	19.67 ± 3.82,153.52 ± 14.34/113.52 ± 5.62 Enalapril 10 mg/d for 8 w (*N* = 34)	1.Hcy, CVCE 2.SBP, DBP	2
10. Wan, 2013	SBP ≥ 140/DBP ≥ 90 mmhg Hcy ≥ 10 μmmol/L	145/149 (294), 62.31 ± 4.16		14.0 ± 12.1,155.2 ± 9.8/93.5 ± 8.2 Folate 0.8 mg + Enalapril 10 mg/d (*N* = 147)	14.0 ± 12.1,152.8 ± 11.1/95.2 ± 9.6 Enalapril 10 mg/d for 8 w (*N* = 147)	1.Hcy 2.SBP, DBP	3
11. Wei, 2013	180 > SBP ≥ 140;110 > DBP ≥ 90 mmhg Hcy ≥ 15 μmmol/L	48/21 (69), 35–73		29.3 ± 4.2, NR Folate 0.8 mg + Enalapril 10 mg/d (*N* = 35)	29.8 ± 3.9, NR Enalapril 10 mg/d (*N* = 34)	1.Hcy 2.CVCE	2
12. Wu, 2013	NR Hcy ≥ 10 μmmol/L	68/32 (100), 56.0 ± 6.0		17 ± 4, NR Folate 0.8 mg + Ealapril 10 mg/d (*N* = 50)	18 ± 5, NR Enalapril 10 mg/d for 12 w (*N* = 50)	1.Hcy 2.CVCE	1
13. Xie, 2013	SBP ≥ 140, DBP ≥ 90 mmHg Hcy ≥ 10 μmmol/L	45/17 (62) 60.1 ± 7.5	48/16 (64) 59.8 ± 6.9	20.1 ± 3.9,152.6 ± 11.7/97.3 ± 8.9 Folate 0.8 mg + Enalapril 10 mg/d (*N* = 62)	19.8 ± 4.1,152.3 ± 12.21/96.81 ± 9.1 Enalapril 10 mg/d for 6 mo (*N* = 64)	1.Hcy 2.SBP, DBP, AE	3
14. Xu, 2013	SBP ≥ 140;DBP ≥ 90 mmHg Hcy ≥ 10 μmmol/L	21/17 (38) 65.84 ± 3.27	19/19 (38) 66.86 ± 3.52	18.35 ± 4.13,159.46 ± 14.32/101.28 ± 7.59 Folate 0.4 mg + Enalapril 10 mg/d (*N* = 38)	17.92 ± 3.93,157.28 ± 13.51/100.32 ± 7.15 Enalapril 10 mg/d for 2 mo (*N* = 38)	1.Hcy, SBP, DBP 2. LVMI, leptin	2
15. Zhang, 2013	NR Hcy ≥ 10 μmmol/L	28/22 (50) 27–81	27/23 (50) 29–80	21.02 ± 4.73,152.59 ± 10.62/95.72 ± 8.52 Folate 0.8 mg + Enalapril 10 mg/d (*N* = 50)	21.11 ± 5.01,153.22 ± 9.86/93.15 ± 8.96 Enalapril 10 mg/d for 2 mo (*N* = 50)	1.Hcy 2.SBP, DBP	1
16. Zhang. H, 2013	SBP ≥ 140/DBP ≥ 90 mmhg Hcy ≥ 10 μmmol/L	41/23 (64), 35–74		NR, 156.8 ± 3.6/107.2 ± 4.3 Folate 0.8 mg + Enalapril 10 mg/d (*N* = 32)	NR, 157.1 ± 4.1/105.5 ± 6.4 Enalapril 10 mg/d for 3mo (*N* = 32)	1.SBP, DBP 2.CVCE	2
17. Zhang. X, 2013	GPCH Hcy ≥ 10 μmmol/L	15/10 (25) 52 ± 8.8	13/12 (25) 51 ± 9.3	NR, NR, Folate 0.4 mg + amlodipine 5 mg/d + benazepril 5 mg/d for 12 w	NR, NR, Amlodipine 5 mg + benazepril 5 mg for 12 w	1.Hcy 2.SBP, DBP	3
18. Zhu, 2013	SBP ≥ 140/DBP ≥ 90 mmhg Hcy ≥ 10 μmmol/L	29/13 (42) 55.8 ± 3.8	33/8 (41) 54.9 ± 3.7	17.51 ± 5.61, 153.31 ± 12.85/92.59 ± 11.28 Folate 0.8 mg + Enalapril 10 mg/d (*N* = 42)	18.15 ± 6.12,152.91 ± 13.12/94.16 ± 12.12 Enalapril 10 mg/d for 12 w (*N* = 41)	1.Hcy, CVCE 2.SBP, DBP	2
19. Feng, 2014	180 > SBP ≥ 140;110 > DBP ≥ 90 mmhg, NR	22/17 (39) 53.3 ± 8.9	23/17 (40) 54.1 ± 9.1	15.6 ± 11.4, NR Folate 0.8 mg + Enalapril 10 mg/d (*N* = 39)	14.9 ± 12.4, NR Enalapril 10 mg/d for 5 w (*N* = 40)	1.Hcy 2.SBP, DBP	2
20. Gao, 2014	NR Hcy ≥ 15 μmmol/L	92/84 (176), 47.5 ± 3.2		NR, NR Folate 0.8 mg + Enalapril 10 mg/d (*N* = 88)	NR, NR Enalapril 10 mg/d for 12 w (*N* = 88)	1.AE 2.SBP, DBP	3
21. Guo, 2014	SBP ≥ 140/DBP ≥ 90 mmhg Hcy ≥ 10 μmmol/L	18/14 (32) 39–75	17/15 (32) 39–74	13.5 ± 12.3,152.3 ± 9.4/94.6 ± 9.1 Folate 0.8 mg + Enalapril1#/d (*N* = 32)	13.6 ± 11.6,153.6 ± 9.8/92.1 ± 7.9 Enalapril 1#/d for 2 mo (*N* = 32)	1.Hcy, CVCE 2.SBP, DBP	2
22. Li, 2014	SBP ≥ 140/DBP ≥ 90 mmhg Hcy ≥ 10 μmmol/L	23/16 (39) 64.1 ± 2.1	27/17(44) 62.9 ± 1.7	17.9 ± 4.9,163.1 ± 10.1/102.56 ± 6.2 Folate 0.8 mg + Enalapril 10 mg/d (*N* = 39)	18.5 ± 4.4,160.7 ± 10.3/101.5 ± 6.4 Enalapril 10 mg/d for 8 w (*N* = 44)	1.SBP, DBP	3
23. Li, J. 2014	180 > SBP ≥ 140;110 > DBP ≥ 90 mmhg Hcy ≥ 15 μmmol/L	47/13 (60) 64.2 ± 7.6	46/14 (60) 63.9 ± 7.9	20.2 ± 4.6,152.4 ± 20.4/92.9 ± 8.8 Folate 0.8 mg + Enalapril1#/d (*N* = 60)	19.9 ± 4.8,153.1 ± 19.6/94.1 ± 8.5 Enalapril 1#/d for 12 mo (*N* = 60)	1.Stroke, AE 2.Hcy, SBP, DBP	3
24. Li, F. 2014	SBP ≥ 140/DBP ≥ 90 mmhg Hcy ≥ 10 μmmol/L	30/26 (56), 53.2 ± 5.6		20.03 ± 3.12,158.3 ± 12.1/98.6 ± 9.0 Folate 0.8 mg + Enalapril 10 mg/d (*N* = 28)	19.72 ± 3.04,157.9 ± 12.1/98.4 ± 9.1 Enalapril 10 mg/d for 12 w (*N* = 28)	1.Stroke, IMT 2.Hcy, SBP, DBP	2
25. Li, X. 2014	SBP ≥ 140/DBP ≥ 90 mmhg Hcy ≥ 10 μmmol/L	59/41 (100), 56.12 ± 8.34		150.2 ± 4.14,150.4 ± 9.0/95.2 ± 6.4 Folate 0.8 mg + Enalapril 10 mg/d (*N* = 50)	14.97 ± 4.96,151.1 ± 8.9/94.6 ± 8.2 Enalapril 10 mg/d for 6 mo (*N* = 50)	1.Hcy, AE 2.SBP, DBP	2
26. Liu, 2014	SBP > 135;DBP > 85mmhg, Hcy ≥ 10 μmmol/L	18/17 (35) 62.0 ± 3.5	NR (35) 55.0 ± 13.5	18.5 ± 4.5,162.8 ± 12.6/106.5 ± 7.5 Folate 0.8 mg + Ealapri l(*N* = 35)	18.9 ± 4.6,161 ± 12.5/106.8 ± 6.9 Enalapril for 8 w (*N* = 35)	1.Hcy, CVCE 2.SBP, DBP	2
27. Lu, 2014	SBP ≥ 140/DBP ≥ 90 mmhg Hcy ≥ 10 μmmol/L	19/13 (32) 58.6 ± 4.9	21/11 (32) 60.1 ± 5.2	20.26 ± 4.63,151.47 ± 10.39/93.69 ± 8.48 Folate 0.4 mg + Enalapril 10 mg/d (*N* = 32)	21.22 ± 5.02,152.19 ± 9.73/91.97 ± 8.86 Enalapril 10 mg/d for 2 mo (*N* = 32)	1.Hcy, CVCE 2.SBP, DBP	1
28. Mu, 2014	SBP ≥ 140/DBP ≥ 90 mmhg Hcy ≥ 10 μmmol/L	62/58 (120), 57.5		21.15 ± 3.78, NR Folate 0.8 mg + Enalapril 10 mg/d (*N* = 60)	20.87 ± 3.81, NR Enalapril 10 mg/d for 12 mo (*N* = 60)	1.Hcy 2. CVCE	1
29. Wang, 2014	SBP ≥ 140/DBP ≥ 90 mmhg Hcy ≥ 10 μmmol/L	38/24 (62), 58		13.7 ± 10.2,159.2 ± 10.9/94.2 ± 8.8 Folate 0.8 mg + Enalapril 10 mg/d (*N* = 31)	13.9 ± 10.0,158.4 ± 10.4/93.6 ± 8.5 Enalapril 10 mg/d for 3 mo (*N* = 31)	1.Hc, AE 2.SBP, DBP	1
30. Xie, 2014	180 > SBP ≥ 140;110 > DBP ≥ 90 mmhg, Hcy ≥ 10 μmmol/L	28/17 (45) 52.7 ± 7.6	29/16 (45) 51.7 ± 7.3	20.39 ± 4.72,153.86 ± 10.47/93.72 ± 8.54 Folate 0.8 mg + Enalapril1#/d (*N* = 45)	21.13 ± 5.03,152.24 ± 9.85/92.86 ± 8.93 Enalapril 1#/d for 8 w (*N* = 45)	1.Hcy, AE 2.SBP, DBP	2
31. Zhang, 2014	SBP ≥ 140/DBP ≥ 90 mmhg Hcy ≥ 10 μmmol/L	39/41 (80) 53.4 ± 9.1	38/42 (80) 51.2 ± 9.3	18.32 ± 8.22, 166.52 ± 9.78/82.96 ± 8.22 Folate 0.4 mg + Enalapril 10 mg/d (*N* = 80)	18.13 ± 7.12, NR Enalapril 10 mg/d for 2 mo (*N* = 80)	1.Hcy	1
32. Chen, 2015	SBP ≥ 140/DBP ≥ 90 mmhg Hcy ≥ 10 μmmol/L	23/19 (42) 63.5 ± 7.3	19/22 (41) 64.2 ± 8.1	12.9 ± 11.8, NR Folate 0.8 mg + Enalapril 10 mg/d (*N* = 42)	13.2 ± 10.6, NR Enalapril 10 mg/d for 8 w (*N* = 41)	1.Hcy	1
33. Hu, 2015	180 > SBP ≥ 140;110 > DBP ≥ 90 mmhg, Hcy ≥ 10 μmmol/L	44/20 (64) 62.1 ± 7.1	43/21 (64) 62.6 ± 7.3	16.1 ± 4.5,156.5 ± 19.7/91.8 ± 8.7 Folate + Enalapril 10 mg/d (*N* = 64)	15.9 ± 4.6,156.1 ± 19.4/92.2 ± 8.2 Enalapril 10 mg/d for 1 y (*N* = 64)	1.Hcy, CVCE 2.SBP, DBP	2
34. Huang, 2015	SBP ≥ 140;DBP ≥ 90 mmHg Hcy ≥ 15 μmmol/L	40/44 (84) 55.8 ± 4.3	50/34 (84) 57.1 ± 5.2	NR,159.8 ± 17.3/88.2 ± 12.7 Folate 0.8 mg + Enalapril 10 mg/d (*N* = 84)	NR,158.6 ± 19.4/90.1 ± 14.6 Enalapril 10 mg/d for 8 w (*N* = 84)	1.Hcy, PWV 2.SBP, DBP, CF	2
35. Jiang, 2015	SBP ≥ 140/DBP ≥ 90 mmhg Hcy ≥ 10 μmmol/L	45/7 (52) 58.2 ± 7.6	40/6 (46) 56.2 ± 8.3	19.3 ± 6.1,149 ± 7/95 ± 4 Folate 0.8 mg + Enalapril 10 mg/d (*N* = 52)	20.0 ± 6.0,147 ± 5/94 ± 3 Indapamide 2.5 mg/d for 3 mo (*N* = 46)	1.P-selectin, TM,Vwf 2. Hcy	2
36. Li, 2015	SBP ≥ 140;DBP ≥ 90 mmHg Hcy ≥ 10 μmmol/L	64/16 (80) 57.12 ± 7.13	60/20 (80) 58.24 ± 6.36	21.16 ± 2.54,169.66 ± 2.54/108.26 ± 2.34 Folate 0.8 mg + Enalapril 10 mg/d (*N* = 80)	20.45 ± 2.26,168.15 ± 1.94/98.45 ± 2.14 Enalapril 10 mg/d for 12 w (*N* = 80)	1.Hcy, SBP, DBP 2. MoCA, HAMD24	2
37. Luo, 2015	SBP ≥ 140/DBP ≥ 90 mmhg Hcy ≥ 10 μmmol/L	50/43 (93) 63.1 ± 7.4	49/43 (92) 64.3 ± 8.3	19.7 ± 6.3,153.5 ± 9.6/94.4 ± 6.9 Folate 0.8 mg + Enalapril 10 mg/d (*N* = 93)	18.8 ± 6.9,152.3 ± 9.1/95.2 ± 7.4 Enalapril 10 mg/d for 24 w (*N* = 92)	1.SBP, DBP 2.BPV, Hcy	2
38. Qin, 2015	SBP ≥ 140/DBP ≥ 90 mmhg Hcy ≥ 10 μmmol/L	57/36 (93) 46 ± 10	53/32 (85) 50 ± 12	20.4 ± 8.5, NR Folate 0.8 mg + anti-hypertension therapy	21.6 ± 9.4, NR Anti-hypertension therapy for 8w	1.Hcy 2.BPV	3
39. Qiu, 2015	NR Hcy ≥ 10 μmmol/L	95/85 (180), 59.3 ± 5.6		20.43 ± 1.23, NR Folate 0.4 mg + Enalapril 5–10 mg/d (*N* = 90)	20.53 ± 1.94, NR Enalapril5- 10 mg/d for 6 mo (*N* = 90)	1.Hcy, MAP 2.MMSE	2
40. Tu, 2015	SBP ≥ 140/DBP ≥ 90 mmhg Hcy ≥ 10 μmmol/L	65/35 (100) 64.2 ± 7.8	61/39 (100) 63.3 ± 8.4	21.7 ± 3.8, NR Folate 0.8 mg + Enalapril 10 mg/d (*N* = 100)	20.6 ± 3.6, NR Enalapril 10 mg/d for 3 mo (*N* = 100)	1.Hcy, MAP 2.CD41, CD62P, CRP	2
41. Wei, 2015	SBP ≥ 140/DBP ≥ 90 mmhg Hcy ≥ 10 μmmol/L	31/18 (49) 56.00 ± 13.13	26/23 (49) 53.50 ± 12.84	14.8 ± 9.4,147.6 ± 11.2/87.4 ± 6.8 Folate 0.8 mg + Enalapril 10 mg.bid/d (*N* = 49)	13.9 ± 9.2,145.7 ± 10.8/84.9 ± 6.5 Enalapril 10 mg.bid/d for 4 w (*N* = 49)	1.Hcy. 2.SBP, DBP	1
42. Xia, 2015	SBP ≥ 140/DBP ≥ 90 mmhg Hcy ≥ 10 μmmol/L	68/32 (100) 62.45 ± 3.28	67/33 (100) 62.08 ± 3.59	18.39 ± 5.01,168.27 ± 12.87/100.07 ± 11.30 Folate 0.4 mg + Enalapril 10 mg/d (*N* = 100)	18.86 ± 5.24,164.24 ± 12.04/95.65 ± 11.83 Enalapril 10 mg/d for 14 d (*N* = 100)	1.Hcy. 2.SBP, DBP	2
43. Zhang, 2015	SBP ≥ 140/DBP ≥ 90 mmhg Hcy ≥ 10 μmmol/L	51/42 (93) 69.18 ± 7.25	53/40 (93) 68.23 ± 6.37	24.12 ± 4.69,155.58 ± 12.74/99.58 ± 8.69 Folate 5 mg + anti-hypertension therapy	24.56 ± 4.63,154.32 ± 11.23/100.74 ± 8.77 Anti-hypertension therapy for 3 mo	1.Hcy, PWV 2.SBP, DBP	2
44. Zhang, X. 2015	SBP ≥ 140;DBP ≥ 90 mmHg Hcy ≥ 10 μmmol/L	60/40 (100) 66.85 ± 5.25	61/39 (100) 67.56 ± 5.73	19.55 ± 3.24,165.75 ± 11.78/110.55 ± 9.28 Folate 5 mg + anti-hypertension therapy	19.75 ± 3.75,163.78 ± 11.75/110.09 ± 9.25 Anti-hypertension therapy for 3 mo	1.Hcy, CVCE	1
45. Zhang, Y. 2015	SBP ≥ 140/DBP ≥ 90 mmhg Hcy ≥ 10 μmmol/L	32/28 (60), 59.7 ± 6.1		20.09 ± 4.13,152.44 ± 10.19/93.24 ± 8.15 Folate + Enalapril1#/d (*N* = 30)	21.98 ± 5.13,152.74 ± 9.37/92.65 ± 8.73 Enalapril 1#/d for 8 w (*N* = 30)	1.Hcy. 2.SBP, DBP	1
46. Zhou, 2015	SBP ≥ 140;DBP ≥ 90 mmHg Hcy ≥ 10 μmmol/L	39/41 (80) 56.89 ± 8.51	38/42 (80) 57.34 ± 7.24	27.86 ± 4.37,160.87 ± 7.91/98.02 ± 7.13 Folate 0.4 mg + Enalapril 10 mg/d (*N* = 80)	28.31 ± 4.51,161.35 ± 8.27/97.28 ± 6.32 Enalapril 10 mg/d for 14 d (*N* = 80)	1.Hcy, CF 2.SBP, DBP	4
47. Bian, 2016	SBP ≥ 140;DBP ≥ 90 mmHg Hcy ≥ 10 μmmol/L	23/21(44) 56 ± 8	21/22 (43) 56 ± 8	18 ± 4,153 ± 10/118 ± 8 Folate 0.4 mg + Enalapril 10 mg/d (*N* = 44)	19 ± 3,152 ± 11/115 ± 7 Enalapril 1# bid/d for 12 w (*N* = 43)	1.Hcy,CVCE 2.SBP,DBP	1
48. Chuan, 2016	SBP ≥ 140;DBP ≥ 90 mmHg Hcy ≥ 10 μmmol/L	80/56 (136) NR	76/54 (130) NR	42.5 ± 9.2,152.1 ± 10.5/92.5 ± 9.2 Folate 0.8 mg + Enalapril 10 mg/d (*N* = 136)	41.9 ± 9.1,150.1 ± 11.2/91.3 ± 10.1 Enalapril 10 mg/d (*N* = 120)	1.Hcy, IMT 2.SBP, DBP	2
49. Huang, 2016	SBP ≥ 140;DBP ≥ 90 mmHg Hcy ≥ 10 μmmol/L	28/12 (40) 72.5 ± 6.9	26/14 (40) 73.8 ± 6.2	24.1 ± 4.6, NR Folate 5 mg + anti-hypertension therapy	23.7 ± 4.1, NR Anti-hypertension therapy for 3 mo	1.Hcy, PWV	1
50. Li, 2016	SBP ≥ 140;DBP ≥ 90 mmHg Hcy ≥ 10 μmmol/L	64/39 (103) ,51.0 ± 9.2		17.83 ± 4.24,158 ± 10/100 ± 7 Folate 0.4 mg + Enalapril (*N* = 52)	17.92 ± 5.04,158 ± 9/101 ± 5 Enalapril 1#/d for 2 mo (*N* = 51)	1.Hcy, 2.SBP, DBP	2
51. Liang, 2016	SBP ≥ 140/DBP ≥ 90 mmhg Hcy ≥ 10 μmmol/L	68/80 (148) 55.8 ± 15.3	70/78 (148) 54.2 ± 14.7	20.7 ± 4.3,153.2 ± 12.5/100.2 ± 5.2 Folate 0.8 mg + Enalapril 10 mg/d (*N* = 148)	21.1 ± 4.1,152.1 ± 10.6/99.1 ± 6.7 Enalapril 10 mg/d for 3 mo (*N* = 148)	1.Hcy, DBP 2.SBP, DBP	2
52. Liu, 2016	SBP ≥ 140;DBP ≥ 90 mmHg Hcy ≥ 10 μmmol/L	34/36 (70) 68.0 ± 8.3	35/35 (70) 69.0 ± 8.1	19.14 ± 4.78,158.32 ± 7.92/78.73 ± 6.54 Folate 0.8 mg + anti-hypertension therapy	19.83 ± 4.32,158.69 ± 8.33/79.15 ± 7.32 Anti-hypertension therapy for 12 w	1.Hcy, IMT 2.SBP, DBP	2
53. Song, 2016	180 > SBP ≥ 140;110 > DBP ≥ 90 mmhg, Hcy ≥ 10 μmmol/L	29/16 (45) 63.13 ± 5.29	31/14(45) 63.59 ± 5.67	15.52 ± 3.24,153.71 ± 6.88/93.72 ± 4.75 Folate + Enalapril1# bid/d (*N* = 45)	16.11 ± 3.54,153.64 ± 5.79/93.19 ± 3.58 Enalapril 1# bid/d for 1y (*N* = 45)	1.Hcy, CVCE 2.SBP, DBP	2
54. Sun, 2016	SBP ≥ 140/DBP ≥ 90 mmhg Hcy ≥ 10 μmmol/L	20/19 (39) 62.4 ± 6.8	21/18 (39) 62.8 ± 7.0	18.11 ± 5.82,151.11 ± 12.65/97.71 ± 11.34 Folate 0.8 mg + Enalapril (*N* = 39)	18.13 ± 5.94,152.10 ± 12.81/97.52 ± 11.87 Enalapril 10 mg/d for 12 mo (*N* = 39)	1.Hcy, CVCE 2.SBP, DBP	2
55. Tang, 2016	SBP ≥ 140;DBP ≥ 90 mmHg Hcy ≥ 10 μmmol/L	39/21 (60) 75.8 ± 8.9	39/21 (60) 73.3 ± 9.2	20 ± 11,157 ± 12/79 ± 8 Folate 0.8 mg + anti-hypertension therapy	19 ± 10,158 ± 12/78 ± 7 Anti-hypertension therapy for 12 w	1.Hcy, CVCE 2.SBP, DBP	3
56. Tian, 2016	SBP ≥ 140/DBP ≥ 90 mmhg Hcy ≥ 10 μmmol/L	48/22 (70) 63.0 ± 6.5	46/24 (70) 65.3 ± 8.3	19.20 ± 4.11, NR, Folate 5 mg tid/d + anti-hypertension therapy	19.25 ± 3.75, NR Anti-hypertension therapy for 6 mo	1.Hcy, CVCE	1
57. Tian, T, 2016	GPCH Hcy ≥ 10 μmmol/L	17/21(38) 58.7 ± 6.3	19/19 (38) 59.2 ± 10.3	18.12 ± 4.78,143.4 ± 12.9/102.4 ± 9.5 Folate + Enalapril 10 mg bid/d (*N* = 38)	17.96 ± 4.67,144.2 ± 13.1/103.6 ± 9.7 Enalapril 10 mg bid/d for 8 w (*N* = 38)	1.Hcy, 2.SBP, DBP	2
58. Wang, 2016	SBP ≥ 140/DBP ≥ 90 mmhg Hcy ≥ 10 μmmol/L	22/20 (42) 59.8 ± 2.7	20/22 (42) 58.6 ± 2.5	17.21 ± 6.21,157.21 ± 10.21/100.81 ± 7.31 Folate 0.4 mg + Enalapril 5 mg bid/d (*N* = 42)	18.47 ± 7.20,158.47 ± 11.20/101.10 ± 6.41 Enalapril 5 mg bid/d for 6 w (*N* = 42)	1.Hcy, 2.SBP, DBP	2
59. Wei, 2016	SBP ≥ 140;DBP ≥ 90 mmHg Hcy ≥ 10 μmmol/L	20/20 (40) 69.86 ± 7.03	22/18 (40) 67.86 ± 5.95	24.7 ± 8.7, NR Folate 0.8 mg + anti-hypertension therapy	23.5 ± 10.6, NR Anti-hypertension therapy for 16 w	1.Hcy, 2.IL-6, MMP-9	2
60. Wu, 2016	SBP ≥ 140/DBP ≥ 90 mmhg Hcy ≥ 10 μmmol/L	19/17 (36) 53.00 ± 5.57	20/16 (36) 55.00 ± 5.13	22.33 ± 4.56,153.77 ± 15.26/112.32 ± 5.55 Folate + Enalapril 5 mg bid/d (*N* = 36)	20.12 ± 4.67,153.42 ± 15.97/114.02 ± 5.77 Enalapril 5 mg/d for 8 w (*N* = 36)	1.Hcy, CVCE 2.SBP, DBP	2
61. Wu. G, 2016	SBP ≥ 140/DBP ≥ 90 mmhg Hcy ≥ 10 μmmol/L	NR (99) 62.3 ± 15.8	NR (97) 62.5 ± 15.1	16.89 ± 5.62,159.51 ± 8.55/98.11 ± 7.44 Folate 0.8 mg + Enalapril10 mg/dl (*N* = 99)	17.11 ± 6.33,160.10 ± 9.12/97.88 ± 7.62 Enalapril 10 mg/d for 10 mo (*N* = 97)	1.Hcy, IMT 2.SBP, DBP	2
62. Yang, 2016	SBP ≥ 140/DBP ≥ 90 mmhg Hcy ≥ 10 μmmol/L	46/34 (80) 66.3 ± 3.4		23.2 ± 9.6,146.4 ± 12.7/85.7 ± 9.4 Folate 0.4 mg + Amlodipine + Benazepril (*N* = 40)	23.0 ± 9.7,147.6 ± 10.8/85.8 ± 9.9 Amlodipine + Benazepril (*N* = 40)	1.Hcy, 2.SBP, DBP	2
63. Zhang, 2016	180 > SBP ≥ 140;110 > DBP ≥ 90 mmhg, Hcy ≥ 15 μmmol/L	29/17 (46) 63.1 ± 2.6	31/15 (46) 65.8 ± 3.5	21.01 ± 3.12, NR Folate 5 mg + Enalapril 10 mg/d (*N* = 46)	20.96 ± 3.05, NR Enalapril 10 mg/d for 8 w (*N* = 46)	1.Hcy, CRP 2.NO, ET-1	3
64. Zhao, 2016	SBP ≥ 140/DBP ≥ 90 mmhg Hcy ≥ 10 μmmol/L	22/20 (42) 59.8 ± 2.7	20/22 (42) 58.6 ± 2.5	17.21 ± 6.21,157.21 ± 10.21/100.80 ± 7.31 Folate 0.4 mg + Enalapril 5 mg bid/d (*N* = 42)	18.47 ± 7.20,158.47 ± 11.20/101.10 ± 6.47 Enalapril 5 mg bid/d for 6 w (*N* = 42)	1.Hcy, CVCE 2.SBP, DBP	2
65. Zhou, 2016	SBP ≥ 140/DBP ≥ 90 mmhg Hcy ≥ 10 μmmol/L	26/22 (48) 62.7 ± 4.8	27/2′ (48) 62.3 ± 4.7	29.34 ± 2.69,162.34 ± 6.96/103.65 ± 3.82 Folate + Enalapril 10 mg/d (*N* = 48)	29.37 ± 2.65,163.28 ± 7.23/102.43 ± 2.96 Enalapril 10 mg/d for 2 mo (*N* = 48)	1.Hcy. 2.SBP, DBP	2

* *GPMH, The guidelines for prevention and management of hypertension (SBP ≥ 140/DBP ≥ 90 mmhg)*.

*SBP, Systolic blood pressure; DBP, Diastolic blood pressure; NR, No report; Hcy, Homocysteine; M/F (n), male/female (numbers); W, weeks; mo, Months; d, Days; IMT, Intima media thickness; CVCE, Cardiovascular and cerebrovascular events; LVMI, Left ventricular mass index; PWV, Pulse wave velocity; CF, Cardiac function; MAP, Mean arterial blood pressure*.

### Meta-analysis of homocysteine lowering therapy on outcomes

**Figure 2:**
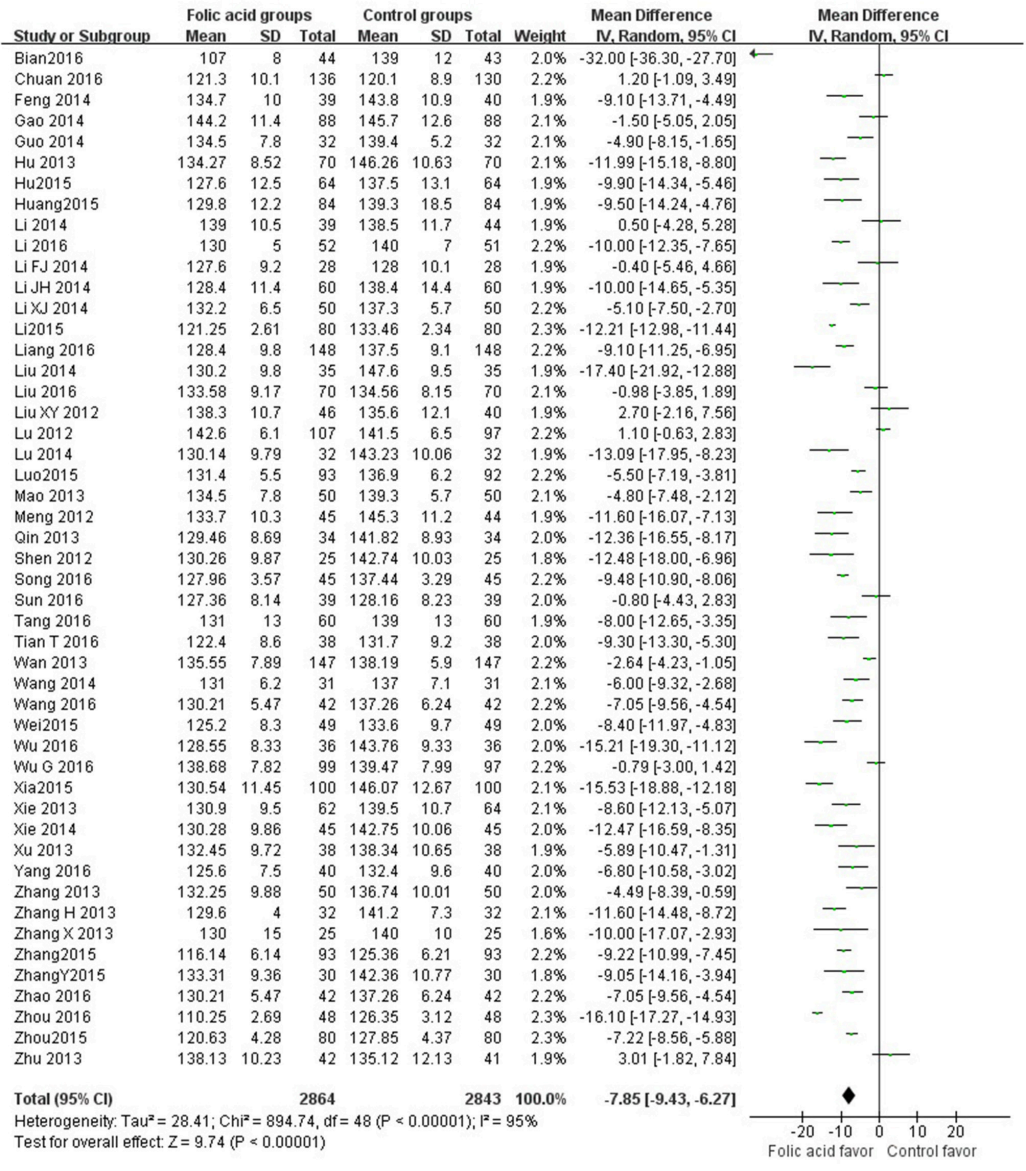
Forest Plot of effect sizes for SBP; SBP: Systolic Blood Pressure.

**Figure 3:**
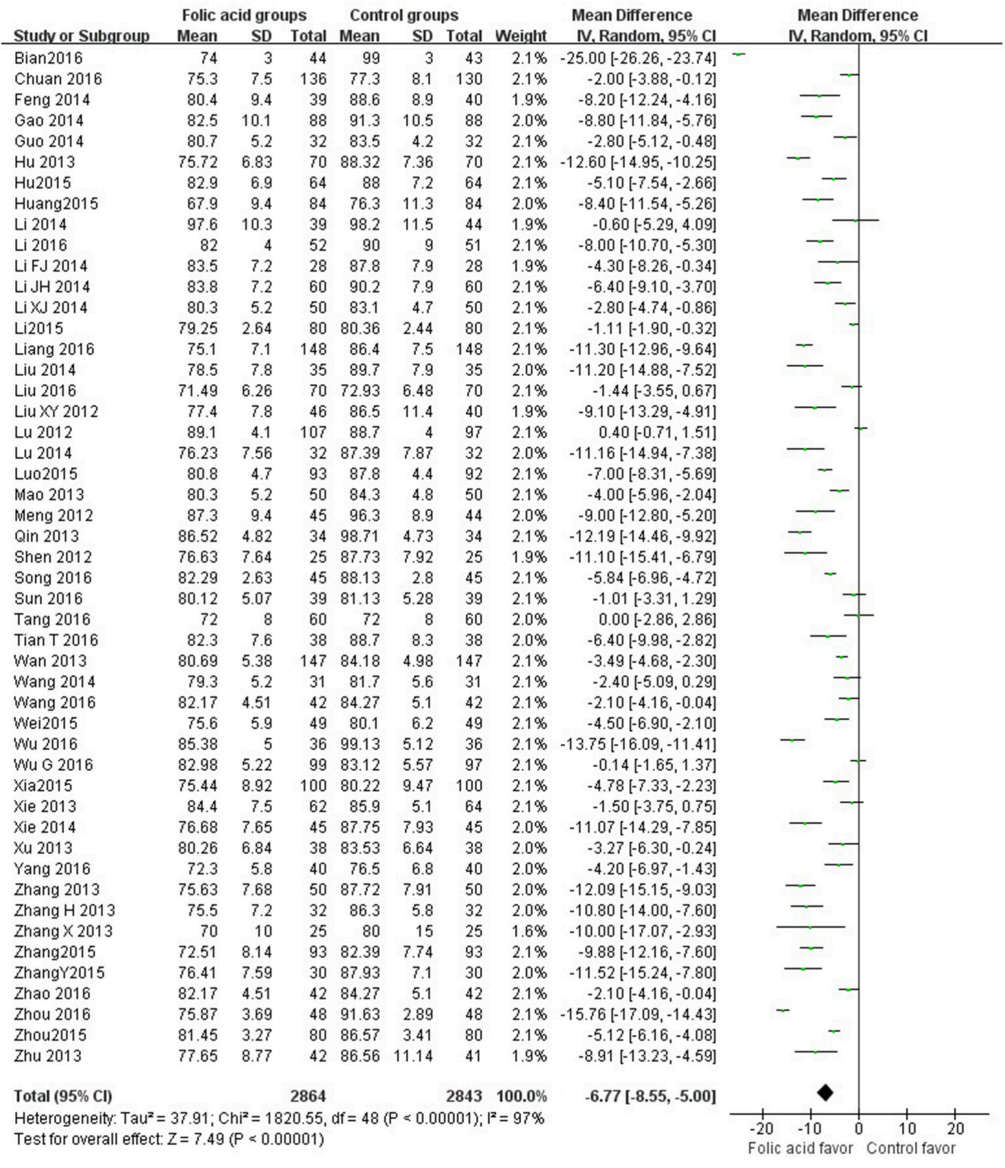
Forest Plot of effect sizes for DBP; DBP: Diastolic Blood Pressure.

**Figure 4:**
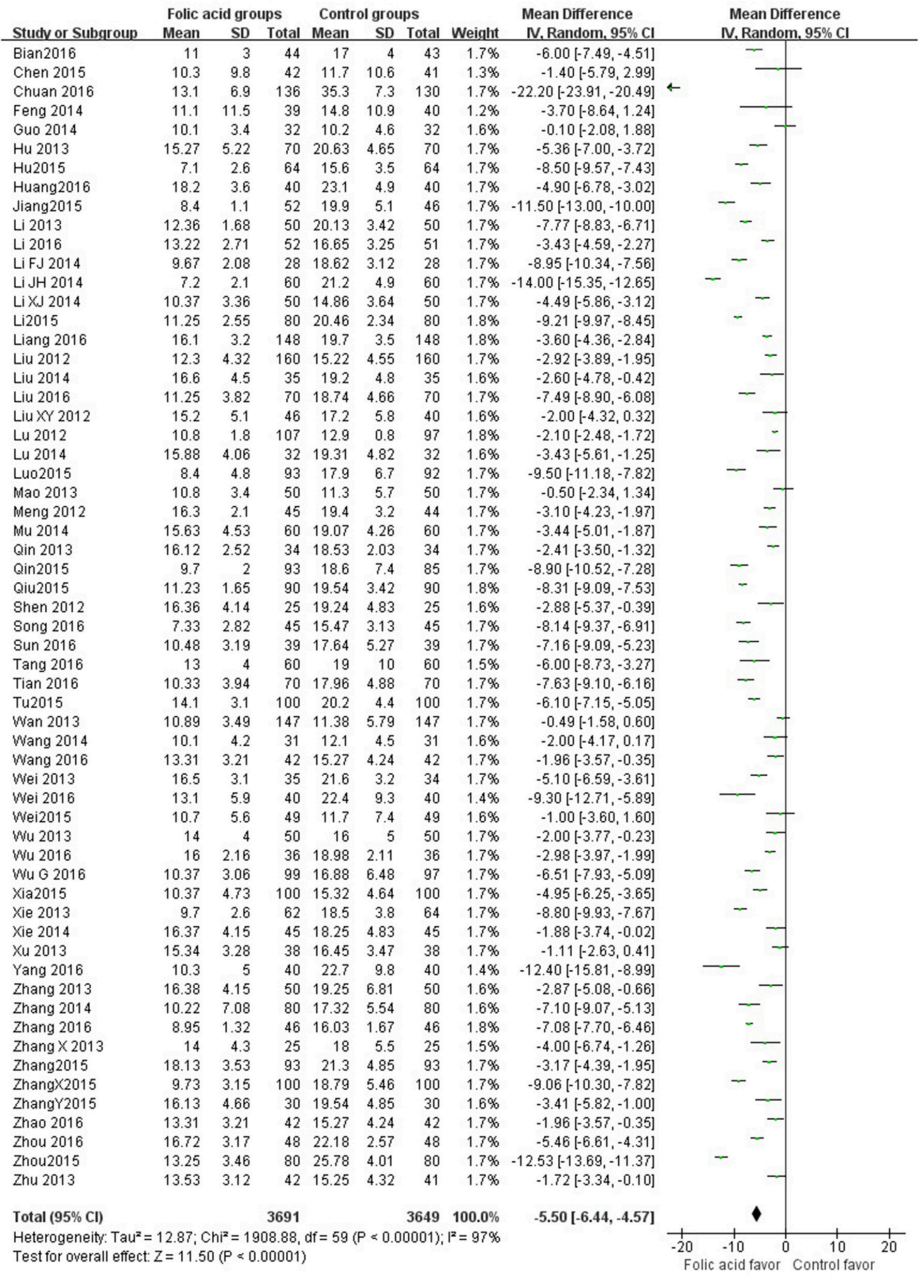
Forest Plot of effect sizes for tHcy level.

**Figure 5:**
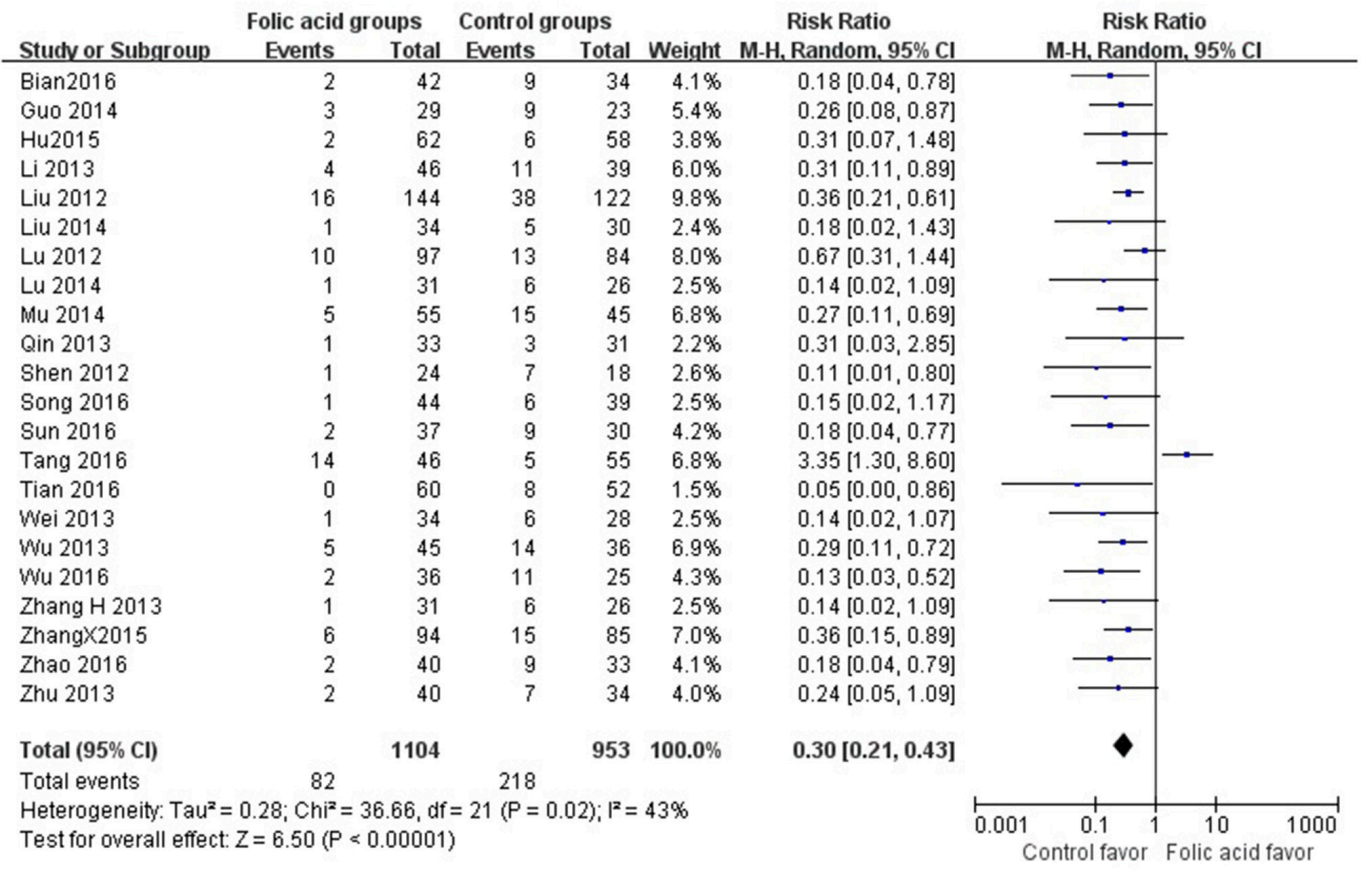
Pooled estimate of decrement in cardiovascular and cerebrovascular events (CVCE).

**Figure 6:**
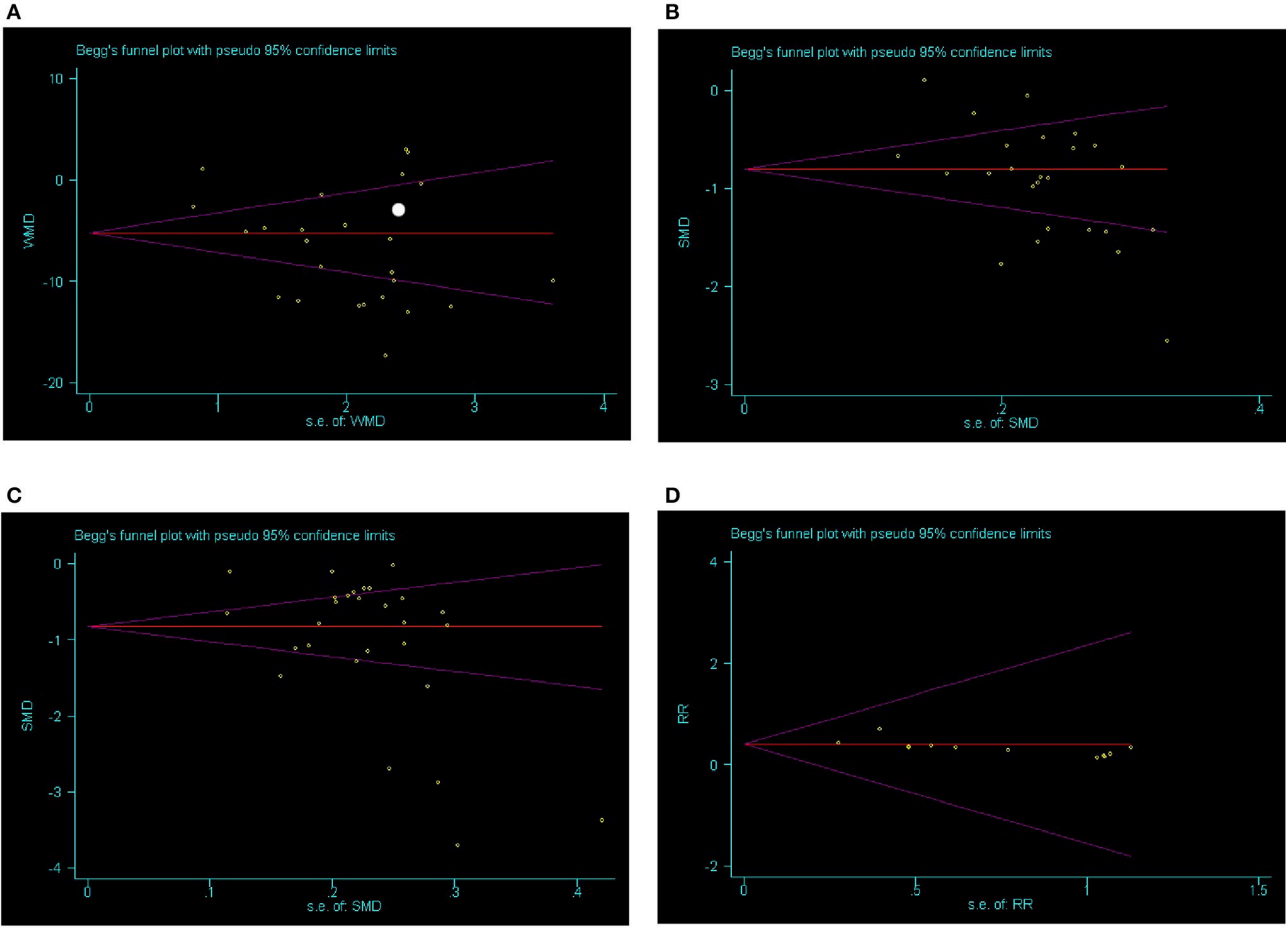
Bias assessment plot for the effect of folic acid supplementation on SBP **(A)**, tHcy levels **(B)**, DBP **(C)**, and CVCE **(D)** by Begg's test.

SBP data were available from 49 trials with 5,707 participants in this analysis of folic acid plus antihypertensive combination therapy compared with antihypertensive alone. We then pooled the whole data to process and found obvious difference between two groups (*p* < 0.00001, WMD = −7.85, 95% CI: −9.43 to −6.27; Heterogeneity test: Tau^2^ = 28.41, Chi^2^ = 894.74, *p* < 0.00001, I^2^ = 95%, Figure 2). Meanwhile, 49 trials displayed significant effect of combination therapy for reducing DBP compared with antihypertensive alone (*p* < 0.00001, WMD = −6.77, 95% CI: −8.55 to −5.00; Heterogeneity test: Tau^2^ = 37.91, Chi^2^ = 1820.55, *p* < 0.00001, I^2^ = 97%, Figure 3). Data on the tHcy levels were available from 60 studies with 7,340 participants included. Compared to antihypertensive alone, the result showed significant effects of folic acid plus antihypertensive therapy for reducing the tHcy levels (*p* < 0.00001, WMD = 5.5, 95% CI: 6.44 to 4.57; Heterogeneity test: Tau^2^ = 12.87, Chi^2^ = 1908.88, *p* < 0.00001, I^2^ = 97%, Figure 4). Due to the high heterogeneity of all analyses, consequently, we should interpret the results prudently (I^2^ = 95%, I^2^ = 97%, I^2^ = 97%, respectively). When pooling the 22 randomized trials with 2,057 subjects, folic acid therapy reduced the danger of CVCE by 12.9% compared with control groups (*p* < 0.00001, RR = 0.30, 95% CI: 0.21 to 0.43; Heterogeneity test: Tau^2^ = 0.28, Chi^2^ = 36.66, *p* = 0.02, I^2^ = 43%, Figure 5). The Begg's tests roughly suggest low likelihood of publication bias for the effect of folic acid on SBP (*p* > 0.05, Figure 6A) and tHcy levels (*p* > 0.05, Figure 6B) rather than DBP (*p* < 0.05, Figure 6C) or CVCE (*p* < 0.05, Figure 6D), respectively. Meanwhile, the funnel plot of the outcome measures by visual inspection were also consistent with the Begg's tests (The Funnel Plots are provided as Supplementary Figure I).

### Stratified analysis

In the stratified analysis by tHcy reduction rate, folic acid fortification and duration of intervention, a greater beneficial effect was observed among those trials of longer intervention duration. The merged RR for the trials with shorter duration was 0.36 (≤12 weeks; 95% CI 0.26 to 0.50, *p* < 0.00001); by contrast the merged RR for the trials with longer duration was 0.31 (>12 weeks; 95% CI 0.22 to 0.43, *p* < 0·00001, Table 2). In addition, when we stratified the included studies by the degree of tHcy lowering, the pooled RR for the studies with a reduction in tHcy concentration of less than 25% was 0.30 (95% CI 0.19 to 0.47 *p* < 0.00001); by contrast, the pooled RR for the trials with a reduction in tHcy concentration of 25% or more was 0.35 (95% CI 0.26 to 0.46, *p* < 0.00001, Table 2). Meanwhile, a bigger beneficial effect was found among those trials of patients with folic acid fortication (RR: 0.27 to 0.18, Table 2). We should explain this result prudently due to only one trial by fortification status, while remaining trials without folic acid fortification.

**Table 2:** Pooled relative risk for CVCE and stratified analysis.

	**CVCE events/total subjects**		**Relative risk (95%CI)**	***P*-value**
	**Folic acid groups**	**Control groups**		
**DURATION OF INTERVENTION**				
>12 weeks	41/569	113/487	0.31 (0.22, 0.43)	*P* < 0.00001
≤12 weeks	40/501	99/438	0.36 (0.26, 0.50)	*P* < 0.00001
**HOMOCYSTEINE LOWERING**				
>25%	58/729	148/635	0.35 (0.26, 0.46)	*P* < 0.00001
≤25%	23/344	64/292	0.30 (0.19, 0.47)	*P* < 0.00001
**FOLIC ACID FORTICATION**				
Yes	3/96	11/88	0.25 (0.07, 0.86)	*P* = 0.03
No	89/905	192/777	0.18 (0.14, 0.40)	*P* < 0.00001

*CVCE, Cardiovascular and cerebrovascular events; CI, Confidence interval*.

## Discussion

### Summary of evidence

In our current meta-analysis, we provided coherent evidence and found apparent benefit of folic acid paratherapy antihypertensive treatments on the risk of cardiovascular and stroke events in primary prevention compared with antihypertensive alone in HT/HHcy. Meanwhile, 65 trials with the total of 7,887 subjects that met the inclusion criteria to process the analysis and the results suggested that folic acid plus antihypertensive combination therapy could evidently reduce the SBP and DBP levels compared to antihypertensive alone. There was also a significance difference in tHcy levels reduction by folic acid supplementation. In addition, in terms of stratified analysis, folic acid fortification and increasing intervention duration showed greater beneficial effects on the risk of CVCE. To our best knowledge from this meta-analysis, we reinforced the efficacy of folic acid plus antihypertensive therapy in HT/HHcy patients.

Our results were consistent with some previous meta-analyses in different subjects (Jardine et al., 2012; Qin et al., 2013). Qin et al. showed that folic acid administration was helpful to reduce CVD risk in patients with kidney disease (Huo et al., 2012). However, Zhou et al concluded that folic acid does not impact on the incidence of major cardiovascular events, myocardial infarction, stroke or all-cause mortality (Zhou et al., 2011). Consequently, we carried out this meta-analysis to explain the possible effect of folic acid on CVCE, SBP and DBP among patients with HT/HHcy.

### Interpretation of the results

Results of previous epidemiologic studies indicated that elevated plasma tHcy levels were popular in the general population and were related to an increased risk for hypertension, CVD and stroke (Eikelboom et al., 1999; Liu and Writing Group of Chinese Guidelines for the Management of Hypertension, 2011). Although the physiological character of hyperhomocysteinemia in multi-organ damage has been suggested, the accurate mechanisms by which it mediates these harmful effects are still unclear but maybe concern on impaired smooth muscle cell function and vascular endothelial (Ganguly and Alam, 2015). Furthermore, hyperhomocysteinemia may be a pivotal pathogenic element in the target organ damages, such as glomerular damage, associated with hypertension, as well as CVD events (Catena et al., 2015). Consequently, management of hyperhomocysteinemia may be helpful to reduce the blood pressure level and the risk of CVCE. However, the clinical meaning of interventions on the tHcy metabolism in the course of the high blood pressure was not fully explored yet. Recently, it had been showed that tHcy levels were associated with SBP and DBP values (Golbahar and Mostafavi, 2012). To our knowledge, insufficient dietary intake of vitamins B, are supposed as a main cause of hyperhomocysteinemia. Hence, attempts to normalize homocystine level by the supplementation of these vitamins, such as folic acid become the main measure (Barnabé et al., 2015). However, Mao et al reported in their study that folic acid in doses 0.4 mg/d and 0.8 mg/d did not lower SBP or DBP, the differences in SBP/DBP change compared to the control group being 0.6/0.0 mmHg (Mao et al., 2008). Moreover, according to the recent report of the China Stroke Primary Prevention Trial (CSPPT) folic acid substitution decreased the risks of stroke without any effect on blood pressure (Huo et al., 2015). All of these results were inconsistent with our findings, We speculated that they were conducted among patient with preexisting cardiovascular disease and none had CVCE as the primary outcome.

### Stratified analyses

Based on the different factors vary the the risk of CVCE. We found that longer duration of intervention seems to more efficacious than shorter duration regarding CVCE in HT/HHcy patients, and the discrepancy between two groups likely to widen with increasing intervention duration. This phenomenon might explain by the pathogenesis of CVCE results from a long and accumulative process, one would anticipant that averting the illness process would also need quite a bit of time (Wang et al., 2007). Our current meta-analysis showed that a decrease of more than 25% in the concentration of homocysteine affected the RR of CVCE slightly more than did a concentration of 25% or less (RR 0.32 vs. 0.31), nevertheless, indicating no significant statistical difference in terms of tHcy lowering level. Which was inconsistent with previous studies showed that an inverse relation between stroke and CVD events and the concentration of tHcy in the blood (Spence et al., 2005). Therefore, the dose-response relation tHcy lowering and CVCE should further validate by much more trials in future. Subgroup analyses of folic acid fortification or not showed the results of fortification had little effect on the risk of CVCE than those without fortification (RR 0.18 vs. 0.27). There were a couple of possibilities which might account for the results. First, in this analysis, only one trial by fortification status, while remaining trials without folic acid fortification, which might also result in certain bias (Xie et al., 2015). Second, folic acid obviously reduced the risk of stroke in countries where grain was not fortified. As any essential nutrient, one would expect that in subjects with ample intake of folic acid, further reduction in the prevalent of stroke would be limited (Wang et al., 2007).

### Limitations

Firstly, a great part of the trials included in this review were of folic acid plus enalapril vs. enalapril, only one trial aim to investigate the difference effect between folic acid plus amlodipine and antihypertensive which did not give the drug, respectively. Secondly, the risk of bias of the included trials was highly variable and not optimal. Nearly all of the included trials had an overall assessment as “high risk of bias” (score 1–3), so we unable to exclude that our results may be biased. Meanwhile, meta-analyses have been often criticized for the potential of publication bias and for the inclusion of poor-quality trials (Rosenthal and DiMatteo, 2001). Thirdly, all the included 65 trials were carried out in China, indicating that there are insufficient data on the US, Africa and Europe. In such a worldwide disease as HT/HHcy, it would inevitable be valuable to know whether the folic acid supplementation is discrepancy in different regions. Interesting, why the addition of folic acid to antihypertensive therapy apparently has only been studied in China. There is striking feature in Chinese that approximate 75% of Chinese hypertension patients are coupled with hyperhomocysteinemia. Therefore, the concept of H-type hypertension was proposed in china (Spence et al., 2005). Then, a large population based studies in China were conducted to test the relation between H-type hypertension and stroke or cardiovascular disease. We have searched the PubMed, Google scholar and CENTRAL et al and found no relevant studies were identified. Fourthly, this meta-analysis also highlights the lack of data on the long term balance of benefits and risks of folic acid supplementation based on the duration of intervention was ranged from 2 to 18 months, as well as the lack of data from multiple large trials that have yet to report results. Finally, CVCE was reported in 22 studies as the outcome measure in this meta-analysis without classifying the detail of subtype. Therefore, we were unable to evaluate the effect of folic acid supplementation on different stroke subtypes (e.g., ischemic or hemorrhagic stroke, fatal or non-fatal stroke) and different cardiovascular disease (e.g., myocardial infarction or stenocardia), because of lack of such data in the published trials (Hu and Xu, 2008). An additional limitation of meta-analyses is that they vary in regard to the characteristics of participants, duration and intensity of treatment, the type of cardiovascular and cerebrovascular event identified, and other design features (Lee et al., 2010). In spite of such limitations, our meta-analysis also had possessed some advantages. Most subjects in this study were not suffered from other disease, such as kidney diseases, diabetes mellitus, thyroid and adrenal glands malfunction, which were exclusion criteria. Furthermore, the body weight, age of patients and the duration of treatments were comparable between two groups. Subjects included in this study without a history of coexisting diseases. Therefore, a large proportion of patients were not treated with other therapies. Regrettably, we did not know the patients whether they were smokers or using any other type of stimulants.

## Conclusions

The findings of this meta-analysis suggested that folic acid supplementation was effective in the primary prevention of CVCE among HT/HHcy patients without a history of vascular diseases, as well as reducing the blood pressure and tHcy levels.

## Author contributions

WW and XW conceived and participated in its design, searched databases, ZZ extracted and assessed studies and helped to draft the manuscript. JH helped in guiding and revising the manuscript. CX participated in the conceptualization and design of the review and revised the review. All authors read and approved the final manuscript.

### Conflict of interest statement

The authors declare that the research was conducted in the absence of any commercial or financial relationships that could be construed as a potential conflict of interest.
