# Supplementary material for: A Meta-Analysis of Folic Acid in Combination with Anti-Hypertension Drugs in Patients with Hypertension and Hyperhomocysteinemia
Source: Front Pharmacol. 2017 Aug 31;8:585. doi: 10.3389/fphar.2017.00585 (PMC5584015; doi:10.3389/fphar.2017.00585)
Supplement: Supplementary file 1 [file DataSheet1.DOC]

**Supplemental Material**

**A meta-analysis of folic acid in combination with anti-hypertension drugs in patients with hypertension and hyperhomocysteinemia**

Wen-Wen Wang^1, a^, Xin-Shi Wang^2, a^, Zeng-Rui Zhang ^2^, Jin-Cai He ^2^, *, Cheng-Long Xie^2^,*

1 The center of Traditional Chinese Medicine, The Second Affiliated Hospital ＆ Yuying Children's Hospital of Wenzhou Medical University, 325027, Wenzhou, China.

2 Department of Neurology, The First Affiliated Hospital of Wenzhou Medical University, Wenzhou 325000, China.

* Correspondence author: Cheng-Long Xie and Jin-Cai He.

Tel/fax: +86 577 8883 2693.

Email: [cl_xie1987@sohu.com](mailto:cl_xie1987@sohu.com) and hjc@wmu.edu.cn.

a These authors contributed equally to this work.

**Supplemental Material I: Full citation details of included studies**

1. Liu YG. The value of enalapril maleate folic acid tablets in prevention H-hypertensive stroke. Guide of china medicine. 2012,10(18):9-10.

2. Liu XY. Qiu ZG. He CX. Clinical observation of enalapril folic acid tablets treat 46 case H type of hypertension. Medical innovation of china. 2012,09(17):12-14.

3. Lu Y. Shen Y. Tang Y. The effects of folic acid on homocysteine levels and incidence of stroke in elder H-hypertension patients. The Journal of Practical Medicine. 2012,28(19):3230-2.

4. Meng X. He YL. The effects of enalapril folic acid tablets in the treatment of H type of hypertension. Chinese Journal of Gerontology. 2012,32(15):3195-7.

5. Shen YJ. Effects analysis of enalapril folic acid on patients with H-type hyperetenbsion. China practical medical. 2012,7(34):156-7.

6. Hu CH. Clinical analysis of enalapril folic acid on patients with H-type hyperetenbsion. Medical information. 2013(25):248-9.

7. Li FR. Xie QG. Enalapril maleate folic acid tablets compared with enalapril tablets in the treatment of H type of hypertension. Practical journal of cardiac cerebral pneumal and vascular disease. 2013,21(9):26-8.

8. Mao HS. Yang MH. Ji B. Xu WL. Clinical analysis of enalapril maleate folic acid tablets on patients with H-type hyperetenbsion. Guide of China Medicine. 2013(22):117-8.

9. Qin YF. Clinical analysis of enalapril maleate folic acid tablets in the treatment of H-type hypertension. Modern Diagnosis & Treatment. 2013(18):4166-7.

10. Wan QH. Huang J. Effects of lowering homocysteine levels between enalapril maleate folic acid tablets compared with enalapril tablets in patients with H-type hypertension. Heilongjiang Medicine Journal. 2013(6):1054-5.

11. Wei XW. Clinical effects of enalapril folic acid tablets on patients with H-type hypertension. Seek medical and ask the medicine. 2013,11(10):300.

12. Wu RY. Li XM. Clinical research of enalapril maleate folic acid tablets treat 100 case H type of hypertension. Chinese Remedies & Clinics. 2013,13(5):669-70.

13. Xie QT. Yang XH. Huang RJ. Effects of enalapril folic acid tablets on homocysteine levels and pulse wave conduction velocity in H-type hypertwnsion patients. China practical medical. 2013,8(20):16-7.

14. Xu TT. Wu Q. Lu XW. Yu F. Tan Y. Effects of Enalapril and Folic on Plasma Levels of Leptin in Patients with h-type Hypertension. Chinese Journal of Experimental Traditional Medical Formulae. 2013,19(10):291-3.

15. Zhang JL. Effects of enalapril folic acid tablets on ambulatory blood pressure in h-type Hypertension patients. Health must read. 2013,12(6):12.

16. Zhang X. Clinical observation of enalapril folic acid tablets in the treatment of patients with H-type hypertension. Chinese-foreign Women's Health. 2013(9):89.

17. Zhang X. Clinical observation of folic acid in the treatment of 50 patients with H-type hypertension. Modern Diagnosis & Treatment. 2013(18):4199-20.

18. Zhu LF. Clinical observation of enalapril folic acid tablets in treating H-type hypertension patirnts. Modern journal of integrated traditional Chinese and western medicine. 2013,22(7):737-9.

19. Feng HJ. Lu P. Chen Q. Clinical Investigation of Application Enalapril Maleate and Folic Acid Tablets Treatment of H-Type Hypertension. Chinese Journal of Medical Guide. 2014(7):1163-4.

20. Gao ZM. Clinical observation of enalapril folic acid tablets treatment of 88 patients with H-type hypertension. Medical Information. 2014(16):523.

21. Guo JK. Clinical effects between enalapril maleate folic acid and enalapril in H-type hypertension patients. Xinxueguanbing Fangzhi Zhishi. 2014(9):28-9.

22．Li J. Effects of folic acid plus enalapril in patients with H-type hypertension. For All Health. 2014(9):272.

23. Li JH. Wang ZG. Observation of enalapril maleate folic acid in treating H-type hypertension patients. Chinese Journal of Integrative Medicine on Cardio Cerebrovascular Disease. 2014(8):1028-9.

24. Li FJ. Effects of folic acid on serum homocysteine levels in H-type hypertension patients. Health Research. 2014(3):303-4.

25. Li XJ. The effects of enalapril maleate folic acid on serum homocysteine levels and blood pressure in H-type hypertension patients. Prevention and Treatment of Cardio-Cerebral-Vascular Disease. 2014(1):76-7.

26. Liu F. Zhang XH. Effects analysis of enalapril folic acid tablets in H-type hypertension patients. Contemporary Medicine. 2014(27):133-4.

27. Lu BF. Observation of enalapril folic acid tablets in treating H-type hypertension patients. Medicine and Health Care. 2014,22(7):81.

28. Mu Y. Effects of enalapril maleate folic acid on blood pressure in H-type hypertension patients. Clinical Medicine. 2014(8):61-2.

29. Wang SR. Comparing two different methods for the patients with H-type hypertension. Chinese Primary Health Care. 2014,28(4):117.

30. Xie ZH. Liu QW. Fang DL. Assessment of enalapril folic acid tablets treatment H-type hypertension patients. Guide of China Medicine. 2014(20):228-9.

31. Zhang LG. Clinical observation of enalapril folic acid tablets in patients with H-type hypertension. Journal of Kunming Medical University. 2014,35(6):154-5.

32.Chen XY. Effects of enalapril maleate folic acid in H-type hypertension patients. Contemporary Medicine. 2015,21(392):115-116.

33.Hu H. Xu ZY. Cheng JM. Li J. Wu QK. The effects of enalapril maleate folic acid in patiernts with H-type hypertension. Guangdong Medical Journal. 2015,36(18):2899-2901.

34.Huang H. Impact of Plymouth Enalapril Maleate Folic Acid Tablets on Arterial Stiffness and Cardiac Diastolic Function in Patients with H-Type Hypertension. J Clin Res. 2015,32(8):1562-1567.

35.Jang XY. Impact of enalapri maleate folic acid tablets on markers of endothelium cell injury and serum homocysteine level inpatients with H - type hypertension. Chin J of Clinical Rational Drug Use. 2015,8(6C):5-7.

36. Li YM. Chen WD. Zhou BJ. Observation of enalapril maleate folic acid on cognitive function and depression scale in H-type hypertension patients. Zhejiang JITCWM. 2015,25(6):560-562.

37. Luo GJ. Ji NN. Ni SM. Clinical research of enalapril maleate folic acid tablets treat 93 case H type of hypertension. Herald of Medicine. 2015,34(3):335-338.

38. Qin JM. Shang XF. Cong XR. Effect of folic acid on homocysteine blood pressure load and blood pressure variability in patients with type H hypertension. China Medicine. 2015,10(12):1753-1756.

39. Qiu LJ. Qiu HQ. Chen SS. Analysis on curative effect of enalapril folic acid tablets and enalapril tablets in the treatment of vascular dementia combined with H type hypertension. Journal of Qiqihar University of Medicine. 2015,36(29):4409-4411.

40. Tu YP. Yao XL. Zen LF. Lei MJ. Wang XH. Gong AB. Wei WR. Wang LL. Ai WW. Wu G. Elevated homocysteine is associated with increased platelet activation in type hypertensive patients：effects of Enalapril／folic acid administration. Chinese Journal of Cardiovascular Research. 2015,13(8):727-730.

41. Wei YH. Clinical observation of enalapril maleate folic acid tablets in H-type hypertension patients. China Prac Med. 2015,10(15):163-164.

42. Xia HB. The effects of enalapril folic acid tablets in the treatment of patients with H-type of hypertension. Medical Information. 2015,28(24):26.

43. Zhang QF. Wang ZY. Xu JP. The effects of folic acid on [atherosclerosis](javascript:void(0);) in elder H-hypertension patients. Chin J Postgrad Med. 2015.38(8):598-600.

44. Zhang XZ. Liu FY. Li J. Wang QQ. Effect of folic acid on plasma homocysteine levels and major cardiovascular events in elderly patients hypertension. Chin J Prim Med Pharm. 2015,22(9):1327-1329.

45. Zhang YC. The effects of enalapril folic acid tablets in H-type Hypertension patients. Drugs and Clinic.2015,23(6):159.

46. Zhou Q. Zhao LY. Li X. Xu XP. Wang BY. Wei CY. Wang JP. Lu XH. Zhang ZM. Ding L. Wang ZQ. Improving effect of enalapril maleate and folic acid tablets on heart function in patients with H-type hypertension and left ventricular hypertrophy. Chin J Evid Based Cardiovasc Med.2016,7(2):188-191.

47. Bian ET. The value of enalapril folic acid tablets in treatment H-hypertensive patients. Guide of china medicine. Drugs and Clinic. 2016,(9):45-47.

48. Quan QY. Zhang Y. Sun J. Cao Y. Effect of Enalapril and Folate Tablet on Carotid Atherosclerotic Plaque in Patients with H-type Hypertension. Chinese Journal of Integrative Medicine on Cardio/Cerebrovascular Disease. 2016,14(15):1702-1704.

49. Huang ZM. Clinical observation of folic acid on homocysteine levels and [atherosclerosis](javascript:void(0);) in elder H-hypertension patients. The northern pharmaceutical. 2016,13(2):36.

50. Lin L. Effects of enalapril folic acid tablets in H-type Hypertension patients. Guide of China Medicine. 2016,14(22):179-180.

51. Liang JM. Luo XM. Xiao L. Huang ZM. Lu D. Clinical assessment of enalapril folic acid tablets treatment H-type hypertension patients. Journal of Taishan Medical College. 2016,37(5):493-494.

52. Liu L. Effects analysis of Low-dose folic acid treatment patients with H-type hypertension. 　Henan Medical Research. 2016,25(6):1004-1005.

53. Song KC. Effect of enalapril maleate folic acid tablets treatment patients with H-type hypertension. Chinese Journal of Integrative Medicine on Cardio/Cerebrovascular Disease. 2016,14(19):2298-2300.

54. Sun F. Wang F. Influence of Enalapril Maleate Folic acid Tablets on blood pressure and serum Hcy leveling patients with H-type hypertension. Journal of North Sichuan Medical College. 2016,31(1):88-90.

55. Tang L. Effect of folic acid supplementation on the levels of plasma homocysteine，hs-CRP and cardiovascular events in elderly patients with type H hypertension. Hebei Medical Journal. 2016,38(7):968-971.

56. Tian JQ. Zen L. The effects of folic acid on homocysteine levels and cardiovascular events in H-hypertension patients. Contemporary Medicine. 2016,22(13):36-37.

57. Tian T. H hypertensive patients using maleic acid enalaprilat folic acid treatment clinical observation. Contemporary Medical Symposium. 2016,14(10):5-6.

58. Wang H. Clinical Analysis of the Treatment of H Type Hypertension Enalapril Maleate and Folic Acid Tablets. Medical Information. 2016,29(15):111-112.

59. Wei N. Shang L. Zhang CF. Yang XL. Impact of folic acid tablets on inflammatory reaction and large arterial elasticity in elderly patients with H - type hypertension. Shanxi medical journal. 2016,45(12):1637-1638.

60. Wu F. Dou YX. Analysis of Effects of Enalaprilat Folic Acid on Treatment of H Hypertension. Journal of Mathematical Medicine. 2016,29(7):1042-1043.

61. Wu GL. Sun YP. Observation of the effect of enalapril folic acid in the treatment of H-type hypertension. Chin J Prim Med Pharm. 2016,23(2):282-284.

62. Yang DY. To Observe the Clinical Effect of Amlodipine Combined with Folic Acid in Treatment of H Type Hypertension. World Latest Medicine Information. 2016,16(25):9-10.

63. Zhang LH. Curative effects of byenalapril combined with folic acid tablets on Hypertension and its mechanism. Journal of Hainan Medical University. 2016,22(2):147-150.

64. Zhou YH. Effects analysis of enalapril and folic acid treatment patients with H-type hypertension. JIN RI JIAN KANG. 2016,15(14):85-86.

65. Zhou KZ. Effects of enalapril folic acid tablets Treatment on H-type hypertension. Patients. Strait Pharmaceutical Journal. 2016,28(9);151-152.

**\**

**Supplemental Figure I: The Funnel Plots of the outcome measures**

**
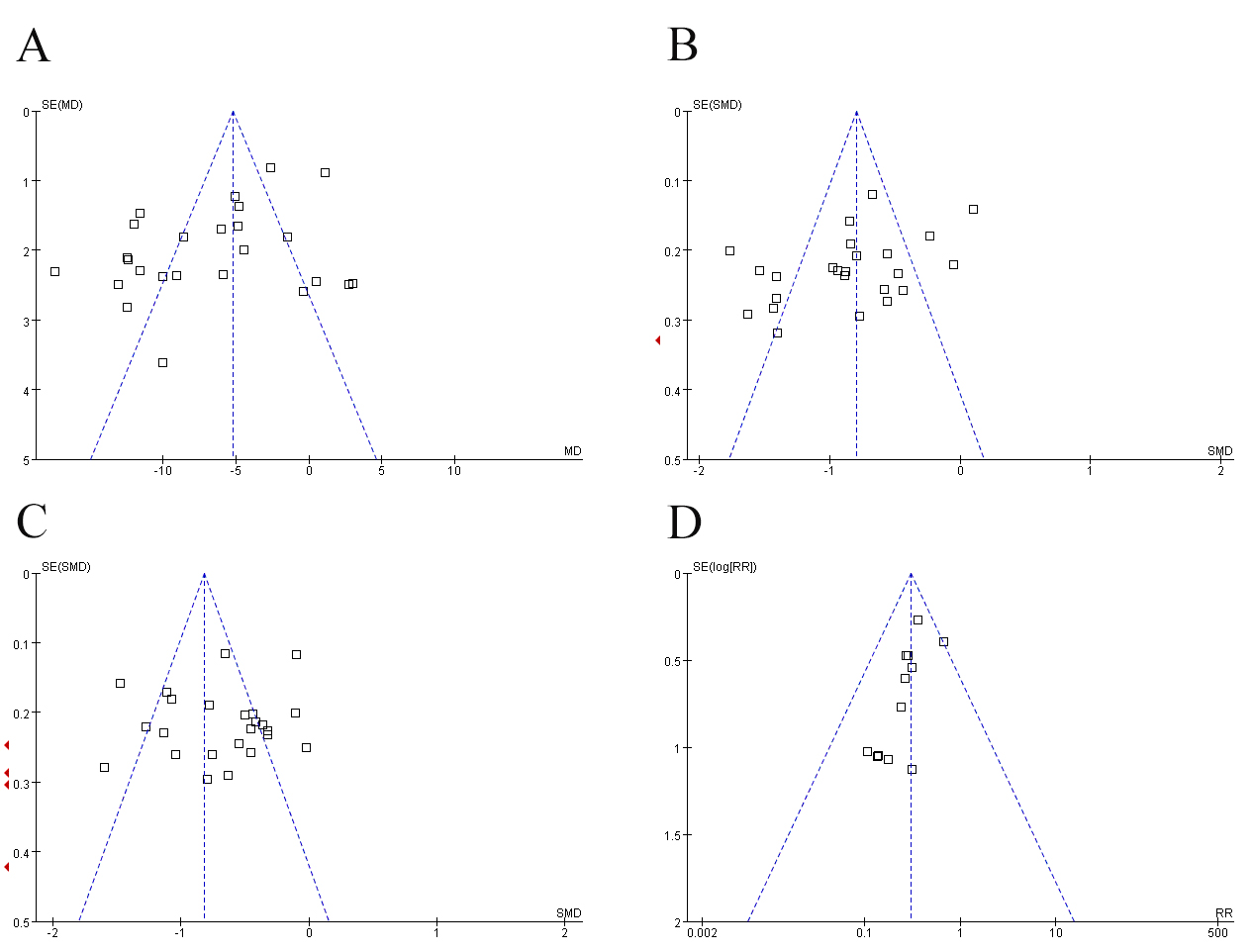
**
